# Providing Pathology Support in Low-Income Countries

**DOI:** 10.1200/JGO.2015.000943

**Published:** 2015-09-23

**Authors:** Shahin Sayed, Robert Lukande, Kenneth A. Fleming

**Affiliations:** Aga Khan University Hospital, Nairobi, Kenya; Makerere University, Kampala, Uganda; University of Oxford, Oxford, United Kingdom

The burden of cancer is on the rise. More than 7 million people per year die as a result of cancer, and it is responsible for more deaths worldwide than AIDS, malaria, and tuberculosis combined. According to the International Agency for Research on Cancer, there were an estimated 14.1 million new cancer cases in 2012. That number is projected to increase to more than 20 million each year by 2030. Nearly 60% of these new cases occur in the developing world with an expected increase to more than 70% in the next 15 years. Despite these alarming statistics, the proportion of the national budget allocated to health in most low- and middle-income countries (LMICs) is less than 5%, contrary to the Abuja Declaration of 2001 in which heads of state of African Union countries^1^ pledged to set a target of at least 15% of the national budget being allocated to improvements in the health sector.

Pathology is a key component in the management of patients with cancer. Pathology as a core discipline in the clinical decision-making process encompasses a broad range of specialties such as histology, cytology, hematology, microbiology, chemical pathology, immunology, and molecular pathology. All have a key role in managing cancer effectively. An accurate diagnosis is critical for the treatment of cancer and clearly has implications in the patient's prognosis and follow-up care. Screening for cancer, assessing margins in tumor excision specimens using frozen sections, determining prognostic and predictive tumor markers, interacting with clinical colleagues in multidisciplinary teams and on tumor boards, and, more recently, interpreting the significance of molecular test results have propelled the traditionally limelight-shy pathologist to the forefront of cancer management.

Despite the critical role of the pathologist in cancer care and the increasing burden outlined in Table 1, pathology in LMICs sadly has been neglected and is struggling to stay relevant. The balance of overstretched resources is tilted toward treatment-intense efforts rather than investing in providing an accurate diagnosis. The economic savings from accurate diagnosis would be sizable and worth the initial expense. Furthermore, accurate data from pathology is the backbone of the cancer registry, which is critical to inform and influence the formulation of health policies at the national and regional level; high-quality cancer registries are limited in LMICs because few treatment centers have adopted standard, internationally acceptable cancer reporting systems. Other problems include the variable standards for training pathologists in LMICs, the scarcity of skilled pathologists (most are overworked and unappreciated), and a lack of appreciation of pathology as a medical specialty in its own right. This lack of understanding of the role of pathology and laboratory physicians by medical health officers and the general public in many LMICs has relegated pathology to the bottom of the specialist ladder. As an example, in most African countries, pathology is perceived to be only a forensic specialty and to be economically unrewarding as a career path for the newly qualified medical graduate. There is fewer than one pathologist per 500,000 people in sub-Saharan Africa (SSA) compared with one pathologist per 15,000 to 20,000 people in the United States and United Kingdom. A 2013 online survey of 34 unique institutions in SSA^2^ that assessed pathology capacity showed that in eight countries, there was no pathologist working within the public sector. In Tanzania, for example, there are only 22 pathologists in the public sector serving a population of 48 million. This scarcity of pathologists in LMICs has in turn encouraged the mushrooming of unregulated laboratories run by poorly qualified non-pathologist staff offering substandard diagnostic services.

**Table 1:** Critical Role of Pathology in Cancer Management in LMICs

LMICs bear more than 60% of current cancer burden in the world, yet in many sub-Saharan African countries, there is less than one pathologist per 500,000 people.
Accurate pathology of cancer determines diagnosis, therapy, prognosis, progression, and response to therapy.
Good-quality pathology is key to saving on limited resources within LMICs.
Accurate and standardized pathology diagnosis is an integral component of quality cancer registries to inform policy in LMICs.
Clinicians in LMICs do not appreciate the role of pathology beyond “malignant” and “benign.” Key to a transformation of the health care workforce, specifically as related to cancer, is better education about the role and repertoire of pathology in the diagnosis and treatment of cancer.

Abbreviation: LMIC, low- to middle-income country.

Amid this often chaotic situation, there are concerted efforts by international partners to help address some of the glaring deficiencies and challenges faced by the profession. Numerous organizations have contributed to pathology training and practice in LMICs over the years, including the Royal College of Pathologists, International Academy of Pathologists, British Division of the International Academy of Pathology, American Society for Clinical Pathology, College of American Pathologists, Friends of Africa, African Organisation for Research and Training in Cancer, and World Association of Societies of Pathology and Laboratory Medicine. Their contributions should not be underestimated. They have supported faculty and trainee exchange programs, shared expertise and best practice standards in cancer diagnostics by providing short courses in LMICs, funded local and regional pathology meetings, and provided assistance in establishing colleges of pathology.

Although the goodwill and the role of international organizations is recognized and appreciated, there is a need to establish an umbrella association that will bring international bodies and their LMIC stakeholders together as equal partners in the areas of training and research in cancer and will also advocate for the profession. This will align with the goal of establishing local sustainable programs and avoid the duplication of effort inherent in the current fragmented approach. One important role of any such body would be to shift the existing practice and culture of identifying LMICs only as sources of research material. The needed paradigm shift would include investing in building local sustainable infrastructure and capacity in research, protecting intellectual property rights for new discoveries, promoting research methodology and independent inquiry, and making research an integral component of medical education. These measures would go a long way toward enhancing future collaborative research within an equal partnership framework and facilitate rapid adoption of new methodology and cancer diagnostics in LMICs.

African Strategies for Advancing Pathology is one such umbrella organization. It helps bring together African pathologists and regional colleges of pathology such as the College of Pathologists of East Central and Southern Africa and the West African College of Pathologists with stakeholders and organizations, including the Association of Pathologists of East Central and Southern Africa, Royal College of Pathologists, British Division of International Academy of Pathology, Friends of Africa, American Society for Clinical Pathology, and International Pathology and Laboratory Medicine Initiative. African Strategies for Advancing Pathology envisions a future in which the importance of pathology in SSA is recognized at local, national, regional, continental, and international levels and in which a competent pathology workforce and several expert centers are supported by a high-quality sustainable delivery system to improve the quality of life of those living in SSA.^3–5^

The private health sector can play a vital role in improving pathology standards and training and can help with integration and cross-transfer of public and private sector skills, knowledge, and expertise to overcome some of the challenges associated with scarce resources in public pathology laboratories.

Creating a culture of quality in pathology laboratories, with emphasis on adoption of quality assurance and proficiency programs, is critical for setting standards in cancer diagnosis. Therefore, this may be an opportune time to establish a stepwise laboratory accreditation system for anatomic pathology laboratories similar to the WHO Stepwise Laboratory Quality Improvement Process Towards Accreditation/Strengthening Laboratory Management Towards Accreditation program in clinical pathology, which has provided a platform for raising the standards of practice and has inculcated a culture of quality in many of the public and private sector clinical pathology laboratories in LMICs. Setting up centers of excellence within a hub-and-spoke model will help ensure quality in pathology practice and, by extension, cancer diagnosis in LMICs and will allow pooling of scarce resources for best practice standards. Lone pathologists practicing in remote, ill-equipped laboratories can be supported through real-time telepathology systems that link to tertiary pathology centers where most pathologists in LMICs are based.

Training non-pathology medical and paramedical staff to perform simple diagnostic techniques such as fine-needle aspiration may overcome some of the challenges associated with the scarcity of pathologists in LMICs. However, there is still a need to train a critical mass of pathologists who can interact with their clinical colleagues and be part of the multidisciplinary team that is crucial for providing quality cancer care.

Along with improving the capacity and skills of pathologists in LMICs, there is a critical need to build skills and competencies for technical staff in anatomic pathology laboratories. Good-quality hematoxylin and eosin tissue sections are an essential component of an accurate diagnosis, and competency in the fundamental principles of tissue fixation and tissue processing for ancillary testing and research need to be embedded in technical curricula. Table 2 summarizes the gaps in cancer diagnosis and pathology in LMICs and suggests some possible solutions and sustainable strategies for addressing short- and long-term needs of patients, training institutions, and national health agendas.

**Table 2:** Challenges, Opportunities, and Proposed Sustainability for Pathology in LMICs for Improved Cancer Care

Issues	Suggested Solutions	Sustainability Plan
Variable standards of training in pathology in LMICs	Harmonize training curriculum through regional and national colleges of pathology for best practice standards. Leverage partnerships with RCPath, BDIAP, ASAP, ASCP, and USCAP for training.	Train and build skills of local faculty.
Poor quality of cancer reports, long turnaround times	Train pathologists to adopt synoptic reporting format for all cancers.	Establish star rating accreditation system for AP laboratories similar to WHO SLIPTA program.
Unregulated practice	Work with technology and/or scientist boards to develop minimum standards for various categories of laboratories.	Sustain advocacy, create public awareness about the role of pathology and pathologists through print and audiovisual media, monitor and evaluate plans to maintain laboratory standards, and work with regulatory bodies.
Overworked pathology staff, poor infrastructure, stock outs leading to substandard AP practice in centers away from major cities	Create centers of excellence using hub-and- spoke model.	Lone practicing pathologists connect to hubs via dynamic real-time telepathology, which can be a platform for training and diagnostic support.
Patients present with advanced-stage disease	Establish the role of the pathologist in cancer screening and early diagnosis.	Strengthen health systems.
Invisible specialty	Encourage multidisciplinary research as a platform for collaboration with clinical colleagues.	Work with international partners to establish consortia for common cancers in LMICs.

Abbreviations: AP, anatomic pathology; ASAP, African Strategies for Advancing Pathology; ASCP, American Society for Clinical Pathology; BDIAP, British Division of International Academy of Pathology; LMIC, low- to middle-income country; RCPath, Royal College of Pathologists, SLIPTA, Stepwise Laboratory Quality Improvement Process Towards Accreditation; USCAP, United States and Canadian Academy of Pathology.

In summary, improving cancer care in LMICs will be undermined if efforts are not focused on strengthening quality in pathology cancer diagnostics. International partners can influence pathology policy and practice in LMICs by pooling scarce resources, participating in collaborative training and research activities, initiating quality assurance programs and, more importantly, advocating for the profession in ministries of health and with the public.

## References

[1] [No authors listed] Abuja Declaration on HIV/AIDS, Tuberculosis and Other Related Infectious Diseases.

[2] Adesina A Chumba D Nelson AM, etal: Improvement of pathology in sub-Saharan Africa Lancet Oncol 14:e152–e157,2013 23561746 10.1016/S1470-2045(12)70598-3

[3] Quality pathology and laboratory diagnostic services are key to improving global health outcomes: Improving global health outcomes is not possible without accurate disease diagnosis Am J Clin Pathol 143:325–328,2015 African Strategies for Advancing Pathology Group Members 25696789 10.1309/AJCP6K0DZCNVCSCI

[4] Proceedings of the African Pathologists Summit: March 22-23, 2013—Dakar, Senegal: A summary Arch Pathol Lab Med 139:126–132,2015 African Pathologists' Summit Working Groups 24963539 10.5858/arpa.2013-0732-CC

[5] Roberts DJ Wilson ML Nelson AM, etal: The good news about cancer in developing countries: Pathology answers the call Lancet 379:712,2012 22364759 10.1016/S0140-6736(12)60306-7

